# Interleukin-17A is a potential therapeutic target predicted by proteomics for systemic sclerosis patients at high risk of pulmonary arterial hypertension

**DOI:** 10.1038/s41598-024-76987-6

**Published:** 2024-11-27

**Authors:** Yuuichi Ono, Akira Mogami, Ryuta Saito, Noriyasu Seki, Sho Ishigaki, Hiroshi Takei, Keiko Yoshimoto, Kenji Chiba, Tsutomu Takeuchi, Yuko Kaneko

**Affiliations:** 1 Sohyaku Innovative Research Division, Mitsubishi Tanabe Pharma Corporation, Yokohama, Kanagawa 227-0033 Japan; 2 Division of Rheumatology, Department of Internal Medicine, Keio University School of Medicine, Tokyo, 160-8582 Japan; 3 Saitama Medical University, Iruma-gun, Saitama 350-0495 Japan

**Keywords:** Systemic sclerosis, Antibody therapy, Target identification

## Abstract

We explored effective therapeutic targets for systemic sclerosis (SSc) patients with high risk for pulmonary arterial hypertension (PAH) by plasma proteomics analysis. A total of fifty-seven patients with SSc were enrolled in the study and the prevalence of PAH was 19.3%. On the other hand, 75.4% of SSc patients showed the ratio of forced vital capacity percentage/diffusing capacity of the lungs for carbon monoxide percentage> 1.6 and met criteria for high risk of PAH. Identification of elevated plasma proteins in SSc patients with high risk of PAH, followed by upstream regulator analysis, predicted interleukin (IL)-17A as a major upstream molecule. Furthermore, we performed in vitro neutralization study using MT-6194, a bispecific antibody targeting both IL-17A and IL-6 receptor. We found that MT-6194 broadly suppressed the increased expression of downstream molecules of IL-17A including IL-17A-related cytokines/chemokines, IL-17A-driven NF$\kappa$B pathway and IL-6-driven JAK/STAT pathway which were shown to be increased in SSc patients with high risk of PAH by the proteomics. Consequently, it is revealed that IL-17A is a promising target for early intervention in SSc patients with high risk for PAH.

## Introduction

Systemic sclerosis (SSc) is an autoimmune disease that includes a wide range of clinical features encompassing vasculopathy, immune dysfunction, and fibrotic manifestations. These features contribute to systemic organ damage and make SSc a life-threatening disease. One of the leading causes of death in SSc patients are SSc-related pulmonary arterial hypertension (SSc-PAH)^1–4^. Early diagnosis and effective therapy for SSc-PAH may lead to improved outcomes^5–7^. Since patients with SSc-PAH are asymptomatic in the early stages, they usually seek medical attention when they begin to experience significant symptoms such as severe exertional dyspnea, dizziness or orthostatic intolerance, angina, or chest pain^8^. A definitive diagnosis of PAH is made through right heart catheterization; however, the prognosis after a confirmed diagnosis is extremely poor, with a 5-year survival rate of around 50%^9^. Under these circumstances, screening algorithms for early diagnosis, such as the DETECT algorithm, have been proposed and are used in clinical practice^10^. Screening patients with SSc-PAH identifies milder forms of the disease, thereby facilitating earlier intervention and improving long-term survival^7^. Therefore, it is suggested that early diagnosis and effective treatment of SSc-PAH can potentially lead to better outcomes.

Research on the pathology of SSc-PAH has progressed, revealing that multiple functions such as endothelial dysfunction, inflammation, immune system activation, and remodeling are involved in pathogenesis, and various biomarkers reflecting these functions have been proposed^11^. Several drugs such as prostacyclin analogs, endothelin receptor antagonists (ERA), and phosphodiesterase inhibitors have been approved for the treatment of PAH, including SSc-PAH patients. However, there has been little progress in improving outcomes^12^. Disease-modifying drugs reflecting the understanding of the disease’s pathology have not yet emerged. The PHAROS study, a prospective longitudinal cohort study observing the natural course of SSc-PAH revealed that SSc patients showing the ratio of forced vital capacity percentage/diffusing capacity of the lungs for carbon monoxide percentage (FVC%/DLCO%)> 1.6 were determined as “at risk” of PAH^13^.

In this study, we focused to analyze the proteomics of SSc patients showing FVC%/DLCO% ratio> 1.6 and having high risk for PAH. Our aim is to understand the characteristics of SSc patients at high risk for PAH through a comprehensive analysis of plasma proteins and to discuss potential drug discovery targets for early therapeutic intervention.

## Results

### SSc patient population

**Fig. 1:**
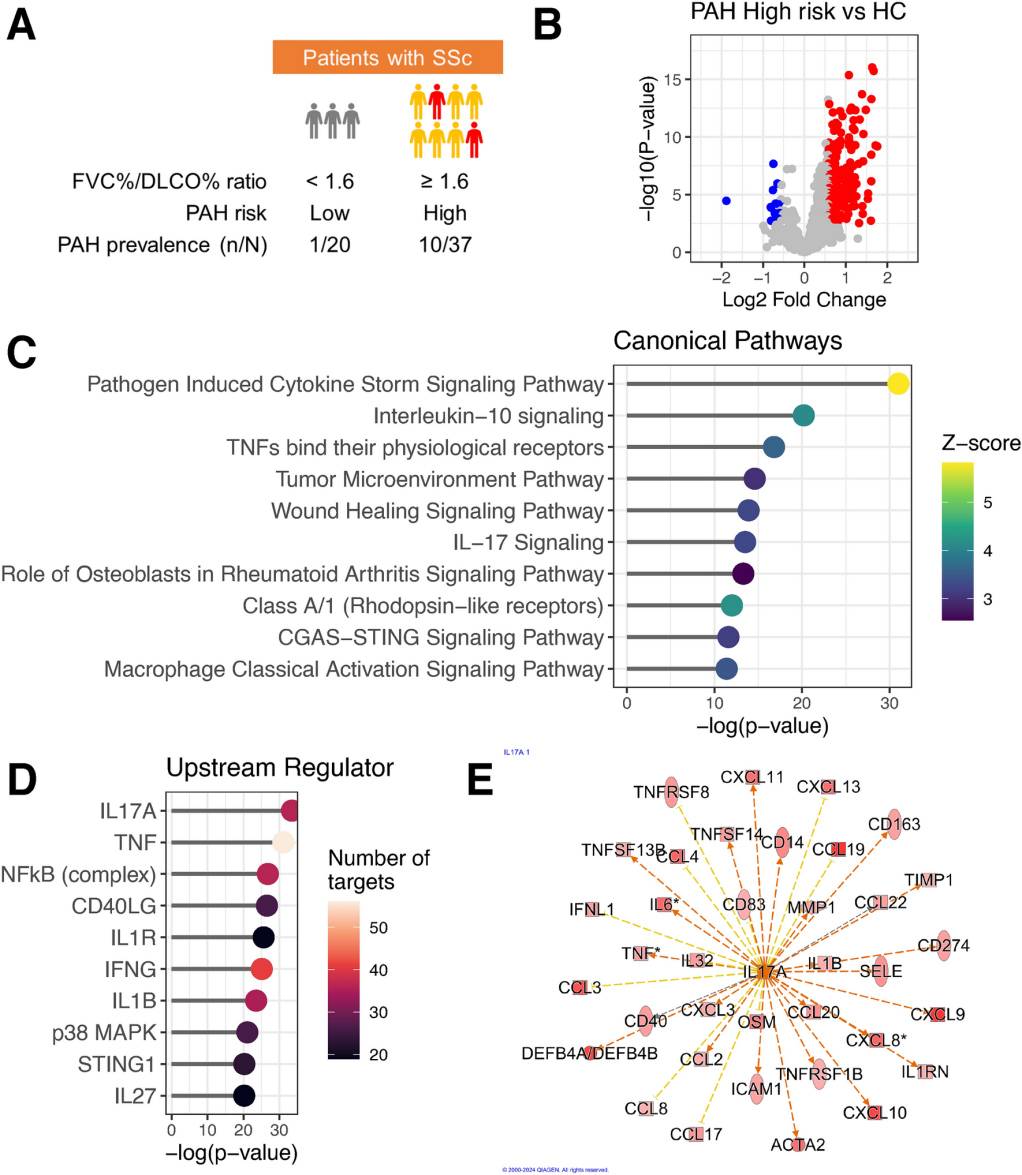
Identification and characterization of differentially expressed proteins in SSc patients at high risk for PAH. (**A**) Patients with an FVC%/DLCO% ratio $\ge$ 1.6 were considered at high risk for PAH. (**B**) Differentially expressed proteins in the plasma of SSc patients at high risk for PAH. Volcano plot visualizing the differences between SSc patients at high risk for PAH and the healthy controls with significance along the y-axis and difference along the x-axis. The significantly up-regulated proteins are marked in red and the significantly down-regulated proteins are marked in blue. Differentially expressed proteins (DEPs) were defined as those exhibiting more than a 1.5-fold change and an FDR < 0.01. (**C**) Canonical pathway enrichment results of DEPs using Ingenuity Pathway Analysis (IPA). The top 10 enriched pathways are shown. The X-axis representing -log(P-value) and the color indicating the z-score, which was calculated based on activity prediction and expression information within the dataset. (**D**) Upstream regulator analysis (top 10 candidates) predicting IL-17A as the leading upstream molecule associated with the elevated proteins in SSc patients at high risk for PAH. The X-axis representing -log(P-value) and the color indicating the number of target molecules. (**E**) IL-17A, predicted upstream regulator, and its downstream molecules were illustrated in a network diagram .

Fifty-seven patients with SSc and 44 healthy controls (HCs) were included in this study. The clinical features of SSc patients are summarized in Table 1. Ages and gender ration of SSc patents and HCs are shown in Supplementary Table 1. Patients with diffuse cutaneous (dc) SSc had more severe skin and lung involvement and were more frequently to have anti-topoisomerase I antibodies. In contrast, patients with limited cutaneous (lc) SSc were more frequently to have anti-centromere antibodies and interstitial lung disease (ILD) but lower modified Rodnan skin scores. The use of medications or the prevalence of other organ involvements showed no significant difference between dcSSc and lcSSc. It has been reported that a ratio of FVC%/DLCO% > 1.6 and DLCO% < 55 are considered a risk factor for the development of SSc-PAH^13^. In our study, 75.4% of patients showed FVC%/DLCO% ratio $\ge$ 1.6 and 64.9% of patients did DLCO% $\le$ 55, though the prevalence of PAH was 19.3%. Noticeably, 10 out of 37 patients showing FVC%/DLCO% ratio $\ge$ 1.6 developed PAH (Fig. 1A). Thus, since more than 70% of SSc patients met the criteria of high risk for PAH, we thought that it is important to understand the features of high-risk patients of PAH in SSc.

**Table 1:** Clinical features of patients with systemic sclerosis. Participant characteristics were summarized using the median with interquartile range (IQR) for continuous variables. Categorial variables are expressed as counts (percentages). For comparison between the dcSSc group and the lcSSc group, Mann–Whitney U-test was used for continuous variables and Fisher’s exact test was used for categorial variables.

Characteristics	SSc (N = 57)	dcSSc (N = 12)	lcSSc (N = 45)	P-value
Age (years)	60.0 (52.0–71.0)	51.0 (44.8–69.5)	64.0 (56.0–71.0)	0.063
Disease duration (years)	7.0 (4.0–16.0)	7.0 (3.8–13.3)	7.0 (4.0–19.0)	0.85
Female sex, n (%)	54 (94.7)	10 (83.3)	44 (97.8)	0.11
Skin involvement				
mRSS	4.0 (3.0–8.0)	12.5 (8.8–16.5)	4.0 (2.0–6.0)	< 0.01
Active digital ulcer, n (%)	6 (10.5)	2 (16.7)	4 (8.9)	0.60
Pulmonary involvement				
FVC (%)	88.1 (78.8–108.9)	72.5 (57.5–84.5)	96.0 (79.8–109.6)	< 0.01
DLCO (%)	47.0 (39.1–61.1)	45.1 (33.9–50.2)	47.7 (41.2–62.0)	0.17
ILD, n (%)	37 (64.9)	12 (100)	25 (55.6)	< 0.01
PAH, n (%)	11 (19.3)	1 (8.3)	10 (22.2)	0.43
FVC%/DLCO%>= 1.6, n (%)	43 (75.4)	7 (58.3)	36 (80.0)	0.14
DLCO% =< 55, n (%)	37 (64.9)	10 (83.3)	27 (60.0)	0.13
Other involvement				
Myocardial involvement, n (%)	16 (28.1)	3 (25.0)	13 (28.9)	> 0.99
Joint involvement, n (%)	7 (12.3)	1 (8.3)	6 (13.3)	> 0.99
Renal involvement, n (%)	2 (3.6)	0 (0.0)	2 (4.4)	> 0.99
Laboratory data				
CRP (mg/dL)	0.05 (0.02–0.14)	0.15 (0.04–0.53)	0.04 (0.02–0.07)	0.065
ANA (U/mL)	640 (320–1280)	1280 (280–2560)	640 (320–1280)	0.17
Anti-centromere antibodies, n (%)	25 (43.9)	1 (8.3)	24 (53.3)	< 0.01
Anti-topoisomerase I antibodies, n (%)	15 (26.3)	8 (66.7)	7 (15.6)	< 0.01
Anti-RNA-polymerase III antibodies, n (%)	4 (7.0)	1 (8.3)	3 (6.7)	> 0.99
Immunosuppressants				
Cyclophosphamide, n (%)	6 (10.5)	3 (25.0)	3 (6.7)	0.10
Azathioprine, n (%)	3 (5.3)	1 (8.3)	2 (4.4)	0.52
Methotrexate, n (%)	2 (3.5)	0 (0.0)	2 (4.4)	> 0.99
Tocilizumab, n (%)	1 (1.8)	0 (0.0)	1 (2.2)	> 0.99
Pulmonary vasodilators				
Calcium channel blockers, n (%)	13 (22.8)	1 (8.3)	12 (26.7)	0.26
Endothelin receptor antagonists, n (%)	7 (12.3)	1 (8.3)	6 (13.3)	> 0.99
Phosphodiesterase 5 inhibitors, n (%)	5 (8.8)	1 (8.3)	4 (8.9)	> 0.99
Prostacyclin, n (%)	17 (29.8)	4 (33.3)	13 (28.9)	0.74
Soluble guanylate cyclase, n (%)	1 (1.8)	0 (0.0)	1 (2.2)	> 0.99

### Differentially expressed proteins in SSc patients with high risk for PAH

To detect proteins increased in the plasma of SSc patients with high risk for PAH, we assessed 1463 proteins utilizing a proximity extension assay (PEA). Differentially expressed proteins were identified in SSc patients with high risk for PAH compared to healthy controls. After adjustment for multiple comparisons using the false discovery rate (FDR), we found 235 up-regulated differentially expressed proteins (Fig. 1B and Supplementary Table 2). The identified proteins included a multitude of inflammatory cytokines and chemokines. To characterize the identified proteins, ingenuity pathway analysis (IPA) was conducted. Figure 1C presents the results of the canonical pathway enrichment analysis (top 10 pathways), with the “pathogen-induced cytokine storm signaling pathway” being predominantly enriched, reflecting inflammation. Figure 1D illustrates the results of the upstream regulator analysis (top 10 candidates), predicting interleukin (IL)-17A as the leading upstream molecule associated with the proteins elevated in patients with high risk of PAH. As upstream molecules, tumor necrosis factor (TNF)-$\alpha$ was predicted alongside IL-17A. It is quite reasonable that nuclear factor-kappa B (NF$\kappa$B) (complex) was expected to be an upstream regulator because NF$\kappa$B signaling pathway is commonly existed in the down-stream of cytokine receptors such as IL-17A, TNF, and IL-1$\beta$ which are the top predictive cytokines. As shown in Fig. 1E, as downstream molecules of IL-17A, we detected IL-17A-related inflammatory cytokines/chemokines such as IL-1$\beta$, TNF-$\alpha$, IL-6, C-C motif chemokine ligand (CCL) 2, and CCL20. Interestingly, we also detected CD163, intracellular adhesion molecule-1 (ICAM-1), IL-32, and IL-6, which are known as biomarkers for SSc-PAH, as downstream molecules of IL-17A. Furthermore, CCL2, C-X-C motif chemokine ligand (CXCL) 8, IL-6, and IL-1$\beta$ were detected as downstream molecules common to IL-17A, TNF, and NF$\kappa$B (Supplementary Fig. 1). The immunological and inflammatory responses play a pathogenic role in SSc-PAH, and subsequently trigger numerous events such as endothelial cell dysregulation and fibroblast activation^11^. Thus, it is highly probable that the downstream molecules of IL-17A are involved in immunological and inflammatory responses in patients at high risk of PAH.

**Fig. 2:**
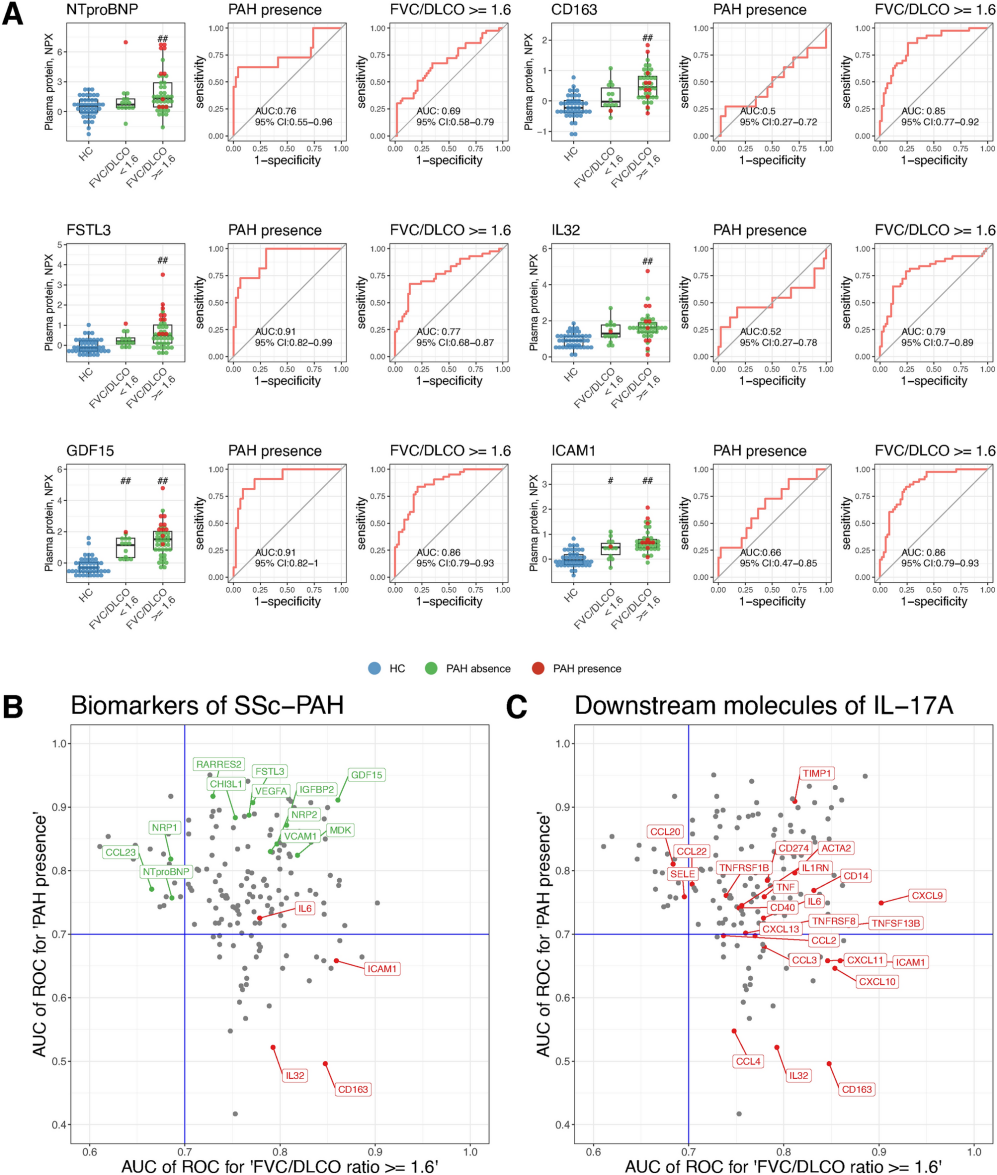
Discriminative performance of differentially expressed proteins associated with FVC%/DLCO% ratio $\ge$ 1.6 and/or the presence of PAH in SSc patients. (**A**) Expression plot of well-known biomarkers of SSc-PAH with receiver operating characteristic (ROC) curves for the presence of PAH and FVC%/DLCO% ratio $\ge$ 1.6. (**B**) Scatter plot of 164 correlated proteins with AUC values from ROC analysis for FVC%/DLCO% ratio $\ge$ 1.6 (X-axis) and PAH presence (Y-axis). Biomarkers for SSc-PAH are highlighted. All of the correlated proteins had high discriminative performance of the presence of PAH and/or FVC%/DLCO% ratio $\ge$ 1.6. (**C**) Scatter plot of 164 correlated proteins with AUC values from ROC analysis for FVC%/DLCO% ratio $\ge$ 1.6 (X-axis) and PAH presence (Y-axis). Downstream molecules of IL-17A are highlighted.

We conducted a similar investigation of SSc patients with an FVC%/DLCO% ratio of less than 1.6 (low risk for PAH) and found upregulated 31 proteins by comparison between SSc patients with low risk of PAH and HCs (Supplementary Fig. 2B and Supplementary Table 3). Canonical pathway analysis confirmed the enrichment of pathways similar to those observed in SSc patients with a high risk for PAH; however, due to the smaller number of DEPs, the Z-scores and -log(p-values) were low, indicating smaller variation (Supplementary Fig. 2C). In SSc patients with low risk for PAH, unlike with high risk, IL-17A was not predicted as an upstream regulator (Supplementary Fig. 2D). Additionally, the predicted downstream molecules for NF$\kappa$B (complex) were less diverse compared to those detected in SSc patients with a high risk for PAH (Supplementary Fig. 2E).

These findings suggest that the enhancement of IL-17A signaling was more pronounced in SSc patients with a high risk for PAH.

### Correlation between differentially expressed proteins and “FVC%/DLCO% ratio $\ge$ 1.6”

Among the identified proteins, we attempted to find proteins that correlated with FVC%/DLCO% ratio $\ge$ 1.6 or the presence of PAH. There were 164 proteins with correlation coefficients greater than 0.4 and p-values less than 0.01 (Supplementary Table 4). Subsequently, receiver operating characteristic (ROC) analyses were performed for both indicators. The identified proteins demonstrated discriminative performance with area under the curve (AUC) > 0.7 for at least one of the metrics. Figure 2A shows the expression levels of several key proteins in SSc patients showing FVC%/DLCO% ratio < 1.6, or $\ge$ 1.6, and in HC, and also displays ROC curves for the presence of PAH or FVC%/DLCO% ratio $\ge$ 1.6. NTproBNP, which reflects cardiac dysfunction, had a high ability to discriminate the presence or absence of PAH. On the other hand, CD163, IL-32, and ICAM-1 had high ability to discriminate FVC%/DLCO% ratio $\ge$ 1.6. Follistatin-related protein 3 (FSTL3) and Growth/differentiation factor 15 (GDF15) showed high performance not only in discriminating the presence of PAH but also in discriminating FVC%/DLCO% ratio $\ge$ 1.6. Figure 2B,C present scatter plots of the AUC for FVC%/DLCO% ratio $\ge$ 1.6 and PAH presence of the 164 identified proteins determined by ROC analysis. The several key molecules of SSc-PAH and downstream molecules of IL-17A are highlighted in Fig. 2B,C, respectively. Regions with an AUC exceeding 0.7 from the ROC analysis for an FVC%/DLCO% ratio $\ge$ 1.6 (top-right and bottom-right quadrants) were enriched with molecules involved in inflammation, vascular remodeling, and endothelial dysfunction. More than 10 downstream molecules of IL-17A are also plotted in the right regions, indicating a potential association with an increased risk of SSc-PAH.

### Increased levels of IL-17A and IL-6 in SSc patients with high risk for PAH

**Fig. 3:**
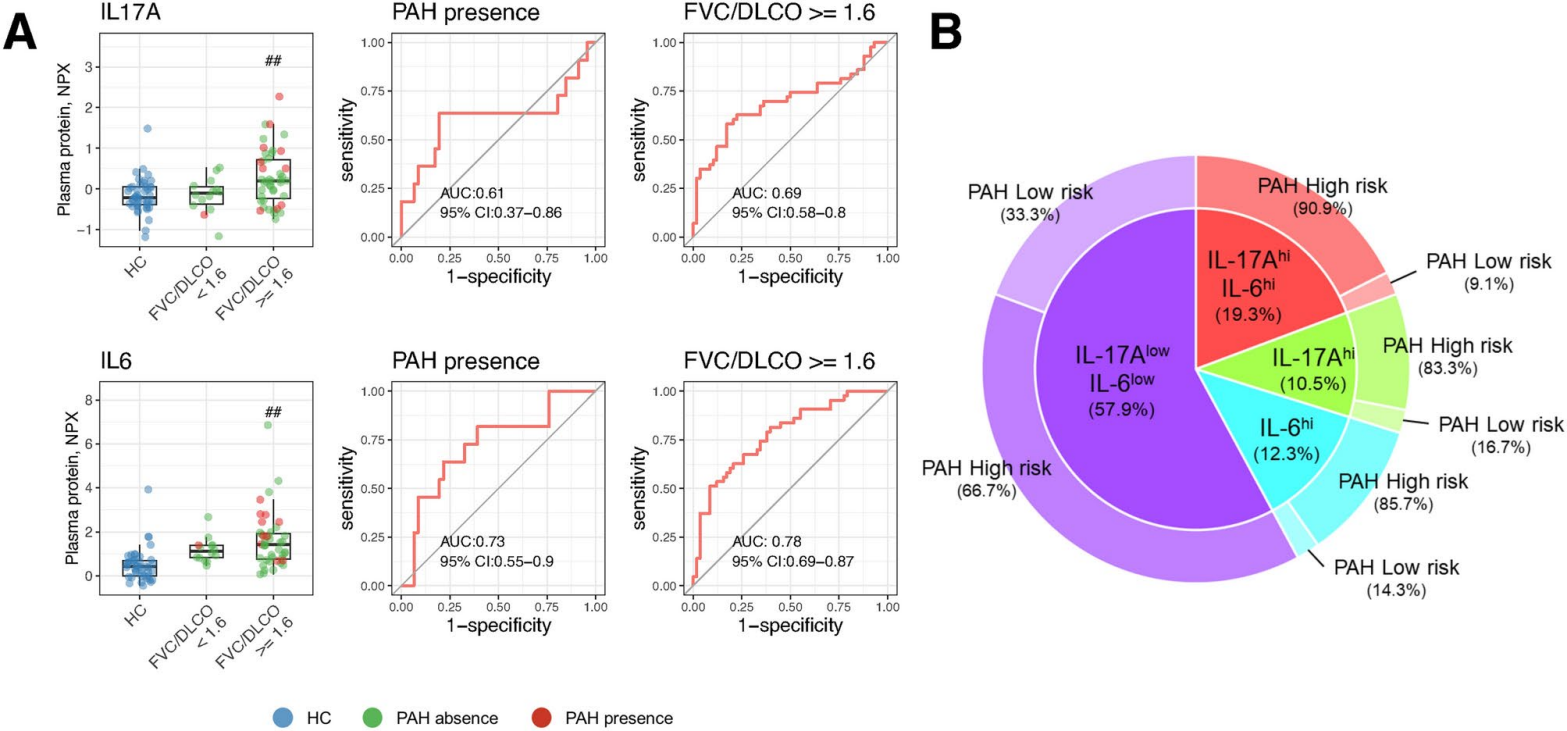
Increased levels of IL-17A and IL-6 in SSc patients at high risk for PAH. (**A**) Expression plot of IL-17A and IL-6 with receiver operating characteristic (ROC) curves for the presence of PAH and FVC%/DLCO% ratio $\ge$ 1.6. Relative quantities of IL-17A and IL-6 in SSc patients stratified by FVC%/DLCO% ratio ($\ge$ 1.6 or < 1.6) compared to healthy controls (HCs). The Y-axis shows the NPX values (Olink’s relative protein quantification unit on a log2 scale). $^{\#\#}$adjusted p-value < 0.01 (FDR): significant difference between the SSc group and the HC group (linear model using limma). (**B**) Proportion of patients with high blood levels of IL-6 and/or IL-17A. Samples exceeding the 95 percentile of the healthy control samples were defined as positive samples. Stratification of SSc samples into four clusters based on serum levels of IL-17A and IL-6. The proportion of patients with FVC%/DLCO% ratio $\ge$ 1.6 in each cluster is shown in the outer ring.

It has been reported that SSc patients with increased serum levels of IL-17A or both IL-17A and IL-6 exhibit a high prevalence of PAH^14^. Additionally, we found in this study that downstream molecules of IL-17A are also associated with high risk for PAH. Therefore, we stratified SSc patients based on FVC%/DLCO% ratio $\ge$ or < 1.6 and examined the levels of IL-17A and IL-6. As shown in Fig. 3A, the levels of IL-17A and IL-6 in the plasma were significantly higher in SSc patients with FVC%/DLCO% ratio $\ge$ 1.6 compared to HCs. Additionally, there were no statistically significant differences of IL-17A and IL-6 between patients with or without immunosuppressants (Supplementary Fig. 4). ROC curve analysis revealed that AUC of IL-17A and IL-6 were 0.61 and 0.73, respectively, between SSc patients with PAH and ones without it. Furthermore, between patients with high risk of PAH and ones without it, AUC of IL-17A and IL-6 were 0.69 and 0.78, respectively. We set the threshold in IL-17A and IL-6 as the 95th percentile value of HC and defined as positive when the cytokine levels were exceeded this threshold. SSc patients were stratified into four clusters based on their serum levels of IL-6 and IL-17A (Fig. 3B). The IL-17A$^{hi}$IL-6$^{hi}$ group comprised patients in which both IL-6 and IL-17A levels exceeded the thresholds. The IL-17A$^{hi}$ group included patients with exceeded thresholds of IL-17A but not IL-6, while the IL-6$^{hi}$ group consisted of patients with exceeded levels of IL-6 but not IL-17A. The IL-17A$^{hi}$IL-6$^{hi}$, IL-17A$^{hi}$, IL-6$^{hi}$, and IL-17A$^{low}$IL-6$^{low}$ groups accounted for 19.3%, 10.5%, 12.3%, and 57.9%, respectively, of the total SSc patients. Subsequently, we examined the proportion of patients showing the FVC%/DLCO% ratio $\ge$ 1.6 in each group. The percentages of patients with high risk of PAH in IL-17A$^{hi}$L-6$^{hi}$, IL-17A$^{hi}$IL-6$^{low}$, IL-17A$^{low}$IL-6$^{hi}$ and IL-17A$^{low}$IL-6$^{low}$ were 90.9, 83.3, 85.7, and 66.7, respectively. These results clearly indicate that patients with high levels of IL-17A or/and IL-6 contain relatively higher frequency of patients with high risk of PAH.

### Blocking of IL-17A and IL-6 signaling reduces the expression of molecules related to high risk for PAH

Because the expressions of IL-17A and its downstream molecules are upregulated in SSc patients with high risk of PAH, targeting these molecules can provide an attractive strategy for drug development. We constructed MT-6194, a bispecific FynomAb targeting both human IL-17A and IL-6R, by genetically fusing the anti-IL-17A Fynomer to the C terminus of the light chain of the anti-IL-6R monoclonal antibody tocilizumab and have been developing this bispecific Ab. (Supplementary Fig. 3)^15^.

**Fig. 4:**
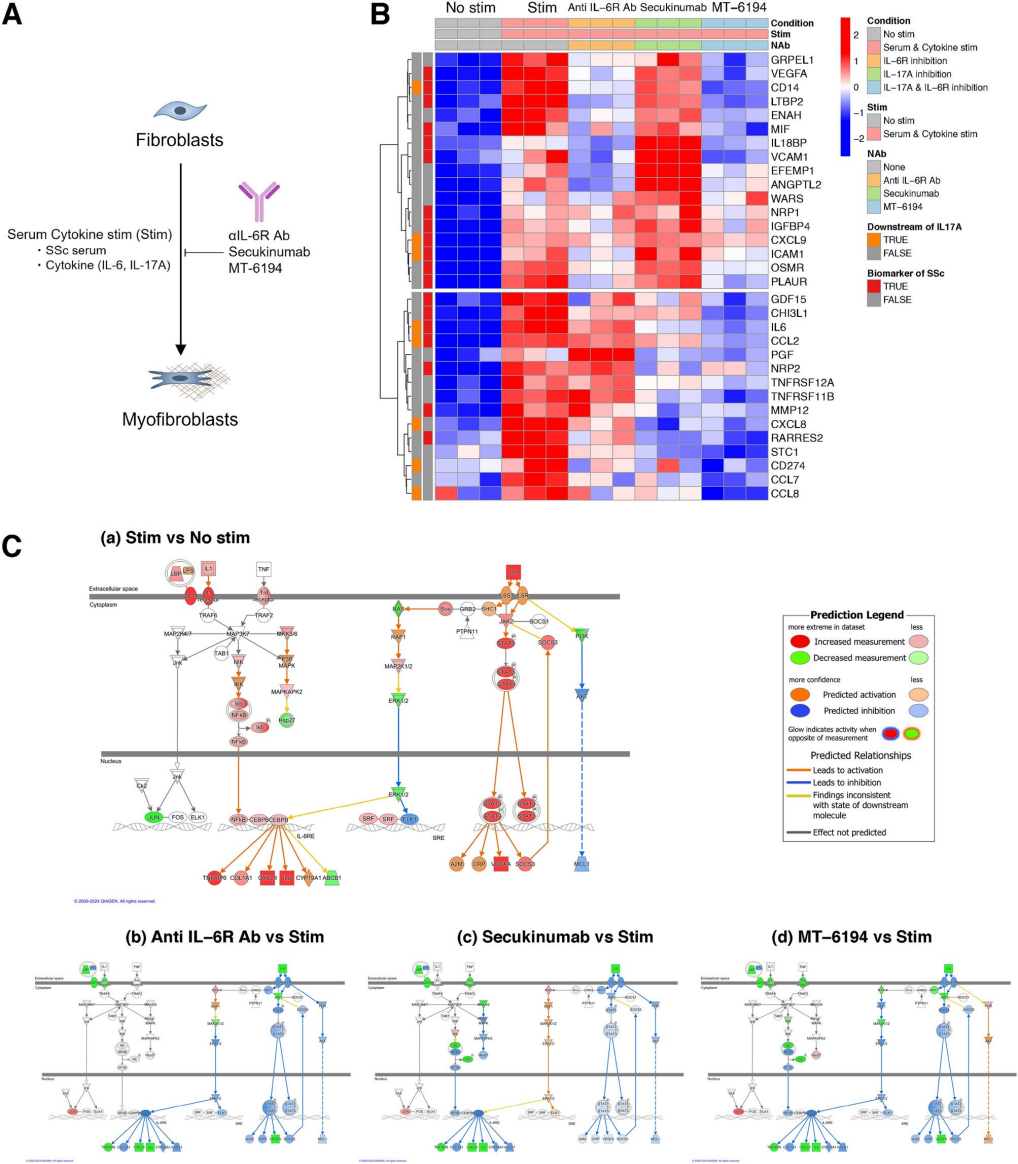
Blocking of IL-17A and IL-6 suppresses the expression of molecules induced by serum cytokines from SSc patients at high risk for PAH. (**A**) Schematic outline of the in vitro neutralization experiment. Normal human dermal fibroblasts were stimulated with serum derived from SSc patients at high risk for PAH, cytokines (IL-17A and IL-6), and IL-6R alpha protein. The neutralizing activities of anti-IL-6R antibody, anti-IL-17A antibody (Secukinumab), and MT-6194 (a bispecific FynomAb targeting both human IL-17A and IL-6R) were evaluated. Gene expression changes were measured using microarray. (**B**) Gene expressions of molecules associated with SSc patients at high risk for PAH. The genes (rows) were rearranged by hierarchical clustering (distance: “Euclidean”; method: “ward”). Sample information was displayed as annotation information in the column direction (see the legend for more details). As row annotations, each gene is color-coded to indicate whether it is a biomarker for SSc or a molecule downstream of IL-17A. (**C**) Overlay of gene expression data onto the IL-6 signaling pathway (an IPA canonical pathway). The NF$\kappa$B pathway is depicted on the left, and the JAK/STAT pathway on the right. Red or green denotes increased or decreased expression based on measured data, while orange or blue represents the predicted outcomes by IPA using the measured data.

To investigate the effects of MT-6194 on the expression of molecules which were upregulated in SSc patients with high risk of PAH, we conducted neutralization studies using normal human dermal fibroblast (NHDF) cells as a model type of cells responding both IL-17A and IL-6 (Fig. 4A). Our preliminary experiments demonstrated that NHDF cells were differentiated into myofibroblasts when cultured in the presence of IL-17A, IL-6, and serum from SSc patients. On the other hand, when the cells were cultured with IL-17A and IL-6 only, we observed the induction of pro-inflammatory cytokine production but not profibrotic activities. Therefore, we can effectively evaluate the neutralizing activities of anti-IL-6R antibody, anti-IL-17A antibody (Secukinumab), or MT-6194 in this assay system. Figure 4B shows the gene expressions of 32 molecules positively correlated with FVC%/DLCO% ratio $\ge$ 1.6 or the presence of PAH. Hierarchical clustering based on gene expressions classified the genes into two major clusters. The upper cluster comprised molecules highly influenced by IL-6R inhibition, including Signal transducer and activator of transcription 3 (STAT3) target molecules such as Vascular endothelial growth factor A (VEGFA). On the other hand, the lower cluster comprised molecules highly influenced by IL-17A inhibition, including target molecules of the NF$\kappa$B pathway such as IL-6 and CXCL8. As expected, MT-6194 broadly suppressed the gene expressions of molecules influenced by IL-6R inhibition or IL-17A inhibition. The molecules influenced by MT-6194 include known biomarkers of SSc (e.g., Matrix metallopeptidase 12 (MMP-12)^16^, CCL2, ICAM-1^17^ and so on) and some of them (e.g., Chemerin, also known as retinoic acid receptor responder protein 2 (RARRES2)^18^, GDF-15, and VEGFA^19^) have been reported to play roles in the pathogenesis of SSc-PAH, indicating that MT-6194 may also impact the pathogenesis of SSc-PAH. These results suggest that dual blocking of IL-6R and IL-17A could more broadly suppress the gene expressions of molecules elevated in SSc patients at high risk of PAH when compared to strategies involving the inhibition of each cytokine individually. Additionally, we also analyzed all the differential expressed genes in the in vitro neutralizing experiments as the heat map image (Supplementary Fig. 5). Similar to the results shown in Fig. 4B, although IL-17A inhibition and IL-6R inhibition resulted in different effects on the gene expressions, MT-6194 affected more gene expressions by inhibiting both IL-17A and IL-6R. The lists of the differentially expressed genes (DEGs) in this study are shown in Supplementary Tables 5, 6, 7, and 8. Figure 4C shows the results of IPA analysis using all DEGs detected in this experiment.

To elucidate the effects of neutralizing antibodies on the activation of the NF$\kappa$B and Janus kinase (JAK)/STAT pathways, gene expression data were overlaid onto the IL-6 signaling pathway, one of the IPA canonical pathways (Fig. 4C). In this pathway diagram, the NF$\kappa$B pathway is depicted on the left and the JAK/STAT pathway on the right, allowing for the visualization of the activation or inhibition of these two pathways. Serum cytokine stimulation activated both the NF$\kappa$B and JAK/STAT pathways. The anti-IL-6R antibody inhibited the JAK/STAT pathway but had no effect on the NF$\kappa$B pathway. Conversely, the anti-IL-17A antibody inhibited the NF$\kappa$B pathway but had no effect on the JAK/STAT pathway. MT-6194 inhibited both the NF$\kappa$B and JAK/STAT pathways. These findings suggest that MT-6194 can broadly suppress the increased expressions of molecules in SSc patients at high risk for PAH by inhibiting both the NF$\kappa$B and JAK/STAT pathways.

## Discussion

Numerous blood biomarkers have been found for the early diagnosis of SSc-PAH and by using these biomarkers, early diagnosis and effective treatment of SSc-PAH may improve outcomes. On the other hand, right heart catheterization is essential for the definitive diagnosis of PAH, however, it is thought that efficient diagnostic tests would be necessary due to its invasiveness. Multidisciplinary approaches, such as the DETECT algorithm, have demonstrated their ability to support the referral of asymptomatic patients for right heart catheterization (RHC) and have proven useful in screening patients with SSc-PAH^10^.

In this study, we investigated the characteristics of SSc patients with high risk for PAH by analyzing changes in plasma proteins, based on the finding that SSc patients with an FVC%/DLCO% ratio $\ge$ 1.6 are at increased risk of developing PAH^13^. Our results clearly indicated that most of the SSc-PAH biomarkers reported to date also exhibited high performance in discriminating an FVC%/DLCO% ratio $\ge$ 1.6. Molecules with high discriminatory ability for an FVC%/DLCO% ratio $\ge$ 1.6 included the proteins related to inflammation, immune response activation, remodeling, endothelial dysfunction, and the pathogenesis of PAH. Noticeably, GDF15, a cytokine of the transforming growth factor (TGF) superfamily, has been associated with tissue injury, inflammation, and vascular remodeling^19^. GDF15 is also known as a biomarker of SSc-PAH with good discriminatory performance, and it has been reported that patients with levels below a threshold of 125 pg/mL have improved survival rates^20^. In contrast, N-terminal pro b-type natriuretic peptide (NTproBNP) exhibited moderate performance in discriminating an FVC%/DLCO% ratio $\ge$ 1.6. NTproBNP, a component of the DETECT algorithm, is also known as a biomarker of PAH that reflects right ventricular (RV) dysfunction^19^. This discrepancy was thought to have occurred because SSc patients at high risk for PAH had not yet reached the stage of right ventricular dysfunction.

To characterize the molecules elevated in SSc patients with high risk for PAH, upstream molecules were predicted using IPA pathway analysis and IL-17A, TNF-$\alpha$, and IL-1$\beta$ were identified. IL-17A exhibits pro-inflammatory and pleiotropic effects on vascular smooth muscle cells, endothelial cells, and fibroblasts, and is associated with vascular damage in systemic sclerosis^21–23^. In addition to its pleiotropic effects, IL-17A has been reported to exhibit synergistic effects with cytokines such as TNF and IL-1$\beta$. The synergistic interactions between IL-17A and TNF-$\alpha$, or IL-17A and IL-1$\beta$, enhance the production of IL-6 and CXCL8 in fibroblasts and endothelial cells, thereby promoting inflammation^24^. Furthermore, since IL-17A is an upstream molecule of TNF-$\alpha$ and IL-1$\beta$, inhibiting IL-17A could efficiently control the effects of these molecules and significantly impact the expression of molecules elevated in SSc patients with high risk for PAH. As noted, our study clearly demonstrated that the various molecules, including IL-17A and its downstream molecules, were significantly increased in SSc patients with high risk for PAH compared to HCs. However, the limitations of this study are relatively small numbers of SSc patients with PAH and are not matched ages and gender ration between SSc patients and HCs. In future, it would be necessary to confirm the results in this study by using lager numbers of SSc patients with PAH.

A treatment framework of PAH centered around pulmonary vasodilators has been established to improve the outcomes of PAH patients. However, the prognosis for SSc-PAH patients is rather poor when compared to those with idiopathic PAH (IPAH) or PAH associated with other connective tissue diseases such as mixed connective tissue disease (MCTD) or systemic lupus erythematosus (SLE). SSc-PAH often coexists with SSc-related ILD (SSc-ILD), latent left ventricular diastolic dysfunction, or pulmonary veno-occlusive disease (PVOD). These comorbidities may lead to reduced tolerance and responsiveness to pulmonary vasodilators in SSc-PAH patients, contributing to their poorer outcomes^25^. Recently, tocilizumab, an anti-interleukin-6 receptor antibody, has been approved as a new therapeutic drug for SSc-ILD in the US. The focuSSced study (phase III randomized controlled trial of tocilizumab for SSc) demonstrated that treatment with tocilizumab was associated with stabilization of the forced vital capacity percent predicted (FVC%) over 48 weeks^26,27^. The approval of tocilizumab for SSc-ILD represents a major expansion of the treatment options. However, there are no disease-modifying drugs approved for SSc-PAH.

By analysis of proteomics data, we found the upregulation IL-17A and its downstream molecules in SSc patients with high risk for PAH prior to the definitive diagnosis of PAH. From these results, we strongly believe that the regulation of IL-17A is an attractive strategy for the treatment of SSc-PAH. As we have been developing MT-6194 that inhibits both IL-17A and IL-6R, we performed in vitro neutralization assay to compare the dual inhibition of both IL-17A and IL-6R with single inhibition of IL-17A or IL-6R. The results of this neutralization revealed that IL-6R inhibition and IL-17A inhibition affected different molecular spectra. Inhibition of IL-6R reduced the gene expression of VEGF through inhibition of the JAK/STAT pathway, while inhibition of IL-17A reduced the expression of IL-6 and CXCL8 through inhibition of the NF$\kappa$B pathway. MT-6194 broadly reduced the gene expressions of the molecules upregulated in SSc patients with high risk for PAH due to simultaneous inhibition of both the JAK/STAT and NF$\kappa$B pathways. These results strongly suggest that dual inhibition of IL-6R and IL-17A can provide an effective therapeutic approach for PAH pathogenesis. In this assay, we used NHDF cells as a model type of cells that are responding with both IL-17A and IL-6 strictly. We confirmed that stimulation with serum of SSc patients increased the gene expressions of several molecules which were upregulated in SSc patients with high risk for PAH. On the other hand, it is thought that endothelial cells or smooth muscle cells than fibroblasts may be more relevant to PAH in SSc patients, if these cells can react with both IL-17A and IL-6. Additionally, it is highly probable that many bioactive molecules are upregulated in serum from systemic sclerosis patients. Thus, we believe that using patient serum as a stimulation condition is crucial to accurately reflect the disease state. Ideally, it would be desirable to conduct experiments using both cells and serum derived from patients. We consider this an important goal for future research.

Since SSc-PAH progresses slowly whereas skin and lung involvements typically appear first, disease-modifying agents that modulate IL-17A and IL-6R are expected to use for the therapy for skin and pulmonary manifestations and may enable the initiation of treatment before the onset of SSc-PAH. This may lead to early therapeutic intervention in SSc-PAH and ultimately contribute to the prevention of SSc-PAH, which is a major cause of SSc-related mortality.

## Methods

### Patient population

Fifty-seven patients with SSc and 44 healthy controls (HCs) were enrolled at Keio University, with written informed consent obtained from all study participants. We reviewed data from patients who visited Keio University Hospital between May 2015 and December 2021 and received a diagnosis of SSc based on the 2013 American College of Rheumatology/European League Against Rheumatism classification criteria for systemic sclerosis^28^. The SSc cohort comprised 12 patients with dcSSc and 45 patients with lcSSc. The HCs were confirmed to be free of any autoimmune diseases, severe allergic disorders, malignancies, or infections. The gender and age distribution of healthy subjects are summarized in Supplementary Table 1 in comparison with SSc patients. The median age of the SSc group was 60 years, with an interquartile range (IQR) of 52.0–71.0 years, and 94.7% of the patients were female. Assessments conducted at the time of sampling encompassed the Modified Rodnan Skin Score (mRSS), the respiratory function tests, the presence of active digital ulcers, interstitial lung disease (ILD), pulmonary arterial hypertension (PAH), and involvement of the joints, renal system, and myocardium. Respiratory function tests were performed, including flow spirometry (forced vital capacity, FVC) and diffusing capacity of the lung for carbon monoxide (DLCO). ILD was defined by the presence of any ground-glass opacity observed on high-resolution computed tomography. PAH was diagnosed when mean pulmonary artery pressure was $\ge$ 25 mmHg with a pulmonary capillary wedge pressure of < 15 mmHg as determined by right-sided heart catheterization, in the absence of significant ILD. Myocardial involvement was identified by the presence of diastolic dysfunction according to Doppler echocardiography diagnostic criteria. Several patients were receiving treatment with immunosuppressants or pulmonary vasodilators at the time of the analysis. All experimental procedures with human subjects followed the institutional research committee’s ethical standards and the 1964 Helsinki Declaration and its later amendments. Approval for this study was granted by the Ethics Committee of Keio University School of Medicine (protocol; 20140335) and by Mitsubishi Tanabe Pharma Corporation (protocol; H14018). For informed consent, we explained the research’s significance, purpose, methods, potential risks, and how samples would be stored and used to all participants, including healthy volunteers, following the explanatory document. We requested their participation and obtained written consent if they agreed. If a proxy was needed, we obtained informed consent from the proxy.

### Plasma protein measurements

Blood samples were collected using Venoject II vacuum blood-collecting vessel (TERUMO, VP-H100K). Following centrifugation at 120 G for 10 min, plasma was aliquoted. The plasma samples were stored at $-80^\circ$C until they were sent to Olink for further analysis. The abundance of 1463 unique proteins in plasma were analyzed using Olink Explore following the standard protocol at Olink Proteomics. Quality control and data normalization according to internal and external controls were carried out using the Olink MyData software. The final assay read-out is given in Normalized Protein eXpression (NPX), which is Olink’s relative protein quantification unit on a log2 scale. For the analysis of the Olink assay, the NPX values were used.

### Neutralization assay using normal human dermal fibroblasts (NHDF)

To investigate the effects of MT-6194, in vitro neutralization experiments with fibroblasts were conducted. Normal Human Dermal Fibroblasts (NHDF; PromoCell, C-12302) reaching approximately 90% confluence were suspended in DMEM containing 10% FBS and seeded at a density of 1.2 $\times$ 10$^5$ cells/0.5 mL per well in a 24-well plate. The following day, the cells were brought to quiescence by incubation in serum-free medium for 24 hours. Subsequently, various stimuli and antibodies were applied. NHDF were stimulated with serum from SSc patients (final concentration: 3%), recombinant human IL-17A (Peprotech, 200-17, 100 ng/mL), recombinant human IL-6 (Peprotech, 200-06, 100 ng/mL), and recombinant human IL-6R alpha protein (R&D Systems, 227-SR, 125 ng/mL) for 2 days. The serum used for stimulation was collected from SSc patients at high risk for PAH. Neutralizing antibodies included human IL-6R alpha antibody (R&D Systems, MAB227), secukinumab, and MT-6194, each at a final concentration of 10 $\upmu$g/mL. After cell culture, cells were lysed with 350 $\upmu$L per well of Isogen II. Total RNA was isolated according to the manufacturer’s instructions. The extracted RNA was dissolved in nuclease-free water and subsequently used for microarray analysis. Gene expression changes were analyzed using the Clariom S human microarray (Thermo Fisher Scientific). All CEL files were loaded into the Applied Biosystems Transcriptome Analysis Console (TAC) 4.0.2 and normalized using the Signal Space Transformation—Robust Multi-chip Analysis (SST-RMA) method. The normalized gene expression data were exported as a CSV file, and subsequent analyses were performed.

### Statistical analysis

All statistical analyses were performed using R (version 4.4.0). For the description of patient population, quantitative variables were expressed as the median with interquartile range (IQR) and categorical variables were expressed as numbers (percentage). To compare lcSSc and dcSSc, Mann Whitney U-test was used for quantitative variables, and Fisher’s exact test was applied for categorical variables. For the differential expression analysis, we utilized the Limma Bioconductor package. Limma is equipped with a comparative analysis algorithm that employs a linear model. We fitted the linear model for all proteins or genes and defined appropriate contrasts to test the hypotheses of interest. To account for multiple testing, significance was assessed using the false discovery rate (FDR). Differentially expressed proteins (DEPs) were defined as those exhibiting more than a 1.5-fold change and an FDR < 0.01 in the proteome analysis. Differentially expressed genes (DEGs) were defined as those exhibiting more than a 1.5-fold change and an FDR < 0.1 in the microarray analysis. Identification of pathways and upstream regulators associated with the DEPs or DEGs was conducted using Qiagen’s Ingenuity Pathway Analysis (IPA, Qiagen, Redwood City, CA, USA). Network figures were generated using IPA. In the pathway analysis, a z-score was utilized, with a threshold of 2 set for significance. The z-score was calculated based on activity prediction and expression information within the dataset. The top 10 pathways with the smallest p-values were displayed in a lollipop plot, with the X-axis representing -log(P-value) and the color indicating the z-score. In the upstream regulators analysis, we focused on molecules predicted to be activated. The top 10 upstream molecules with the smallest p-values were displayed in a lollipop plot, with the X-axis representing -log(P-value) and the color indicating the number of target molecules. Additionally, the predicted upstream regulators and their associated downstream molecules were illustrated in a network diagram. The gene expression data from microarray analysis were overlaid onto the “IL-6 signaling” network figure registered in the IPA canonical pathway. As indicated in the prediction legend, red or green denotes increased or decreased expression based on measured data, while orange or blue represents the predicted outcomes by IPA using the measured data. To perform correlation analysis between categorical variables and continuous variables of NPX, we calculated the point-bispecific correlation coefficients for the following 2 aspects: “whether FVC%/DLCO% $\ge$ 1.6 is met” or “whether PAH is present”. We focused on DEPs with correlation coefficients of more than 0.4 and p-values of less than 0.01. The discriminative performance of DEPs was assessed by receiver operating characteristic (ROC) analyses for both indicators. Area under the curve (AUC) was recorded as an index of discriminative accuracy and compared among DEPs. The identified DEPs demonstrated discriminative performance with area under the curve (AUC)> 0.7 for at least one of the metrics. Among the molecules elevated in scleroderma patients at high risk of PAH, the genes that showed increased expression in the in vitro NHDF assay upon stimulation were used for clustering analysis. Unsupervised hierarchical clustering of gene expression (rows) was performed using Euclidean distance and Ward linkage methods. A heat map was created based on the expression level of each gene. Expression levels were scaled to mean expression level of 0 and standard deviation of 1. As row annotations, each gene is color-coded to indicate whether it is a biomarker for SSc or a molecule downstream of IL-17A. The samples were placed in columns, and the experimental conditions (presence or absence of stimulation and type of neutralizing antibody) were labeled by color coding with column annotation.

## Supplementary Information

Supplementary Information 1.

Supplementary Information 2.

Supplementary Information 3.

Supplementary Information 4.

Supplementary Information 5.

Supplementary Information 6.

Supplementary Information 7.

Supplementary Information 8.

Supplementary Information 9.

Supplementary Information 10.

## Data Availability

Data will be made available on request. Correspondence and requests for materials should be addressed to Kenji Chiba.
