# Supplementary material for: Interleukin-17A is a potential therapeutic target predicted by proteomics for systemic sclerosis patients at high risk of pulmonary arterial hypertension
Source: Sci Rep. 2024 Nov 27;14:29484. doi: 10.1038/s41598-024-76987-6 (PMC11603215; doi:10.1038/s41598-024-76987-6)
Supplement: Supplementary file 1 — Supplementary Information 1. [file 41598_2024_76987_MOESM1_ESM.pdf]

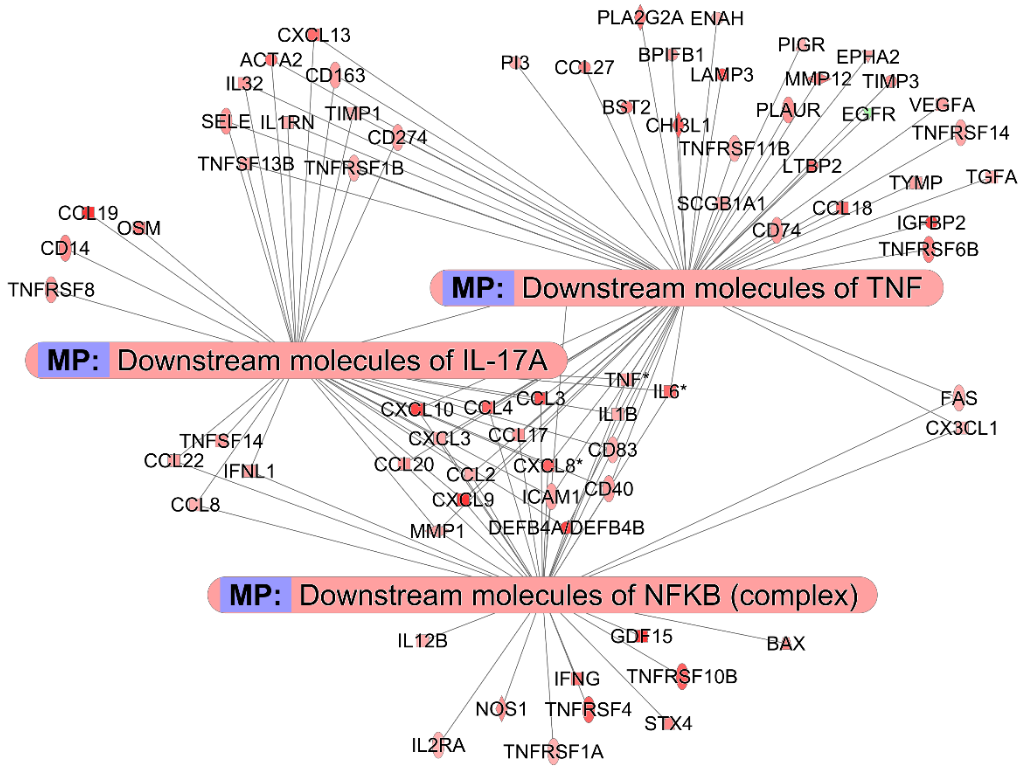

Supplementary Figure 1 The top 3 upstream molecules shared downstream molecules.

The top 3 upstream molecules (IL-17A, TNF, NFκB complex) and their downstream molecules were shown in network diagrams. Some downstream molecules were common to multiple upstream molecules.

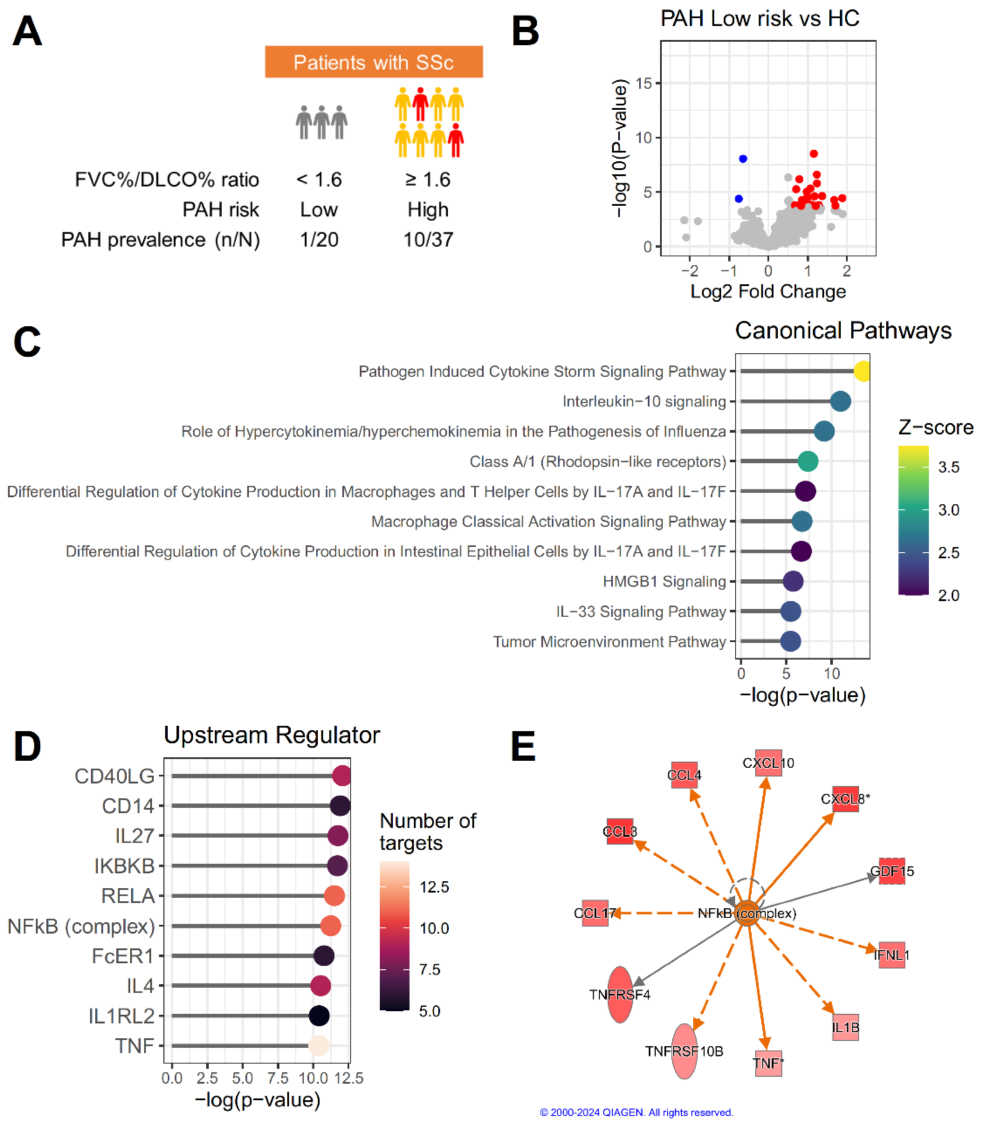

Supplementary Figure 2 Identification and characterization of differentially expressed proteins in SSc patients at low risk for PAH.

(A) Patients with an FVC%/DLCO% ratio < 1.6 were considered at low risk for PAH. (B) Differentially expressed proteins in the plasma of SSc patients at low risk for PAH. Volcano plot visualizing the differences between SSc patients at low risk for PAH and the

healthy controls with significance along the y-axis and difference along the x-axis. The significantly up-regulated proteins are marked in red and the significantly down-regulated proteins are marked in blue. Differentially expressed proteins (DEPs) were defined as those exhibiting more than a 1.5-fold change and an FDR < 0.01. (C) Canonical pathway enrichment results of DEPs using Ingenuity Pathway Analysis (IPA). The top 10 enriched pathways are shown. The X-axis representing -log(P-value) and the color indicating the z-score, which was calculated based on activity prediction and expression information within the dataset. (D) Upstream regulator analysis (top 10 candidates). The X-axis representing -log(P-value) and the color indicating the number of target molecules. (E) NFκB complex, predicted upstream regulator, and its downstream molecules were illustrated in a network diagram.

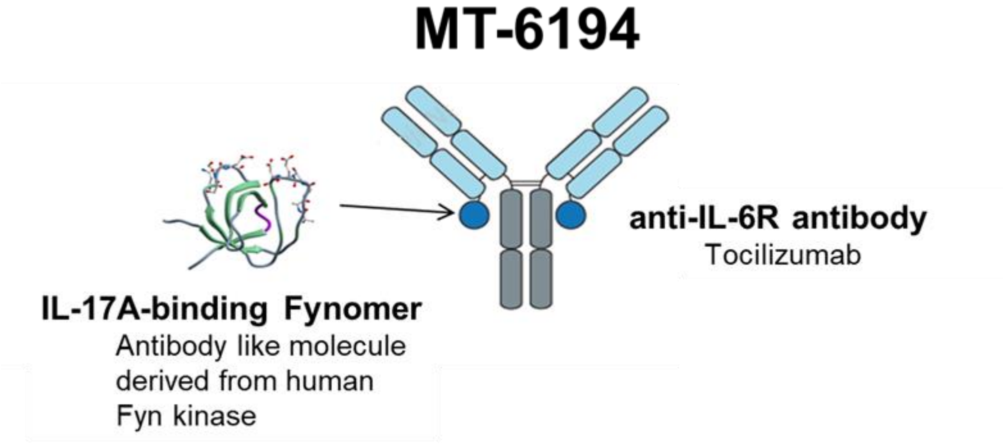

Supplementary Figure 3 Scheme of the structure of MT-6194.

MT-6194, a bispecific FynomAb targeting both human IL-17A and IL-6R, was constructed by genetically fusing the anti-IL-17A Fynomer to the C terminus of the light chain of the anti-IL-6R antibody tocilizumab. MT-6194 consistently had a similar affinity as tocilizumab for human IL-6R and a higher affinity for human IL-17A than secukinumab.

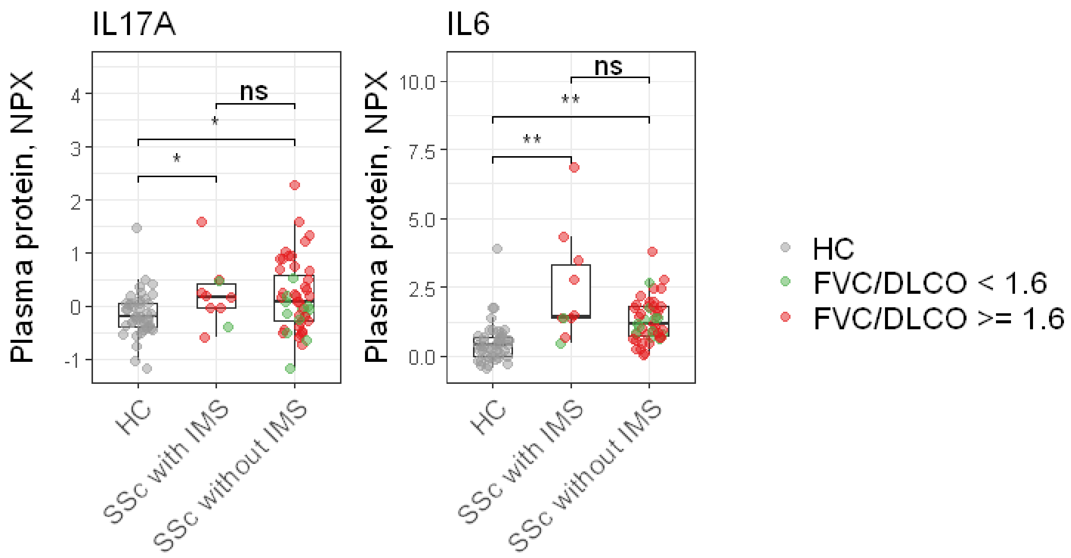

Supplementary Figure 4 The levels of plasma IL-17A and IL-6 in SSc patients treated with immunosuppressive drugs (IMS) or not treated. （HC n=44, SSc patients treated with IMS n=10, SSc patients with no IMS n=47）

The results are expressed as the median with interquartile range (IQR) and statistical differences were analyzed by Mann Whitney U-test (*P < 0.05, **P < 0.01, ns: not significant).

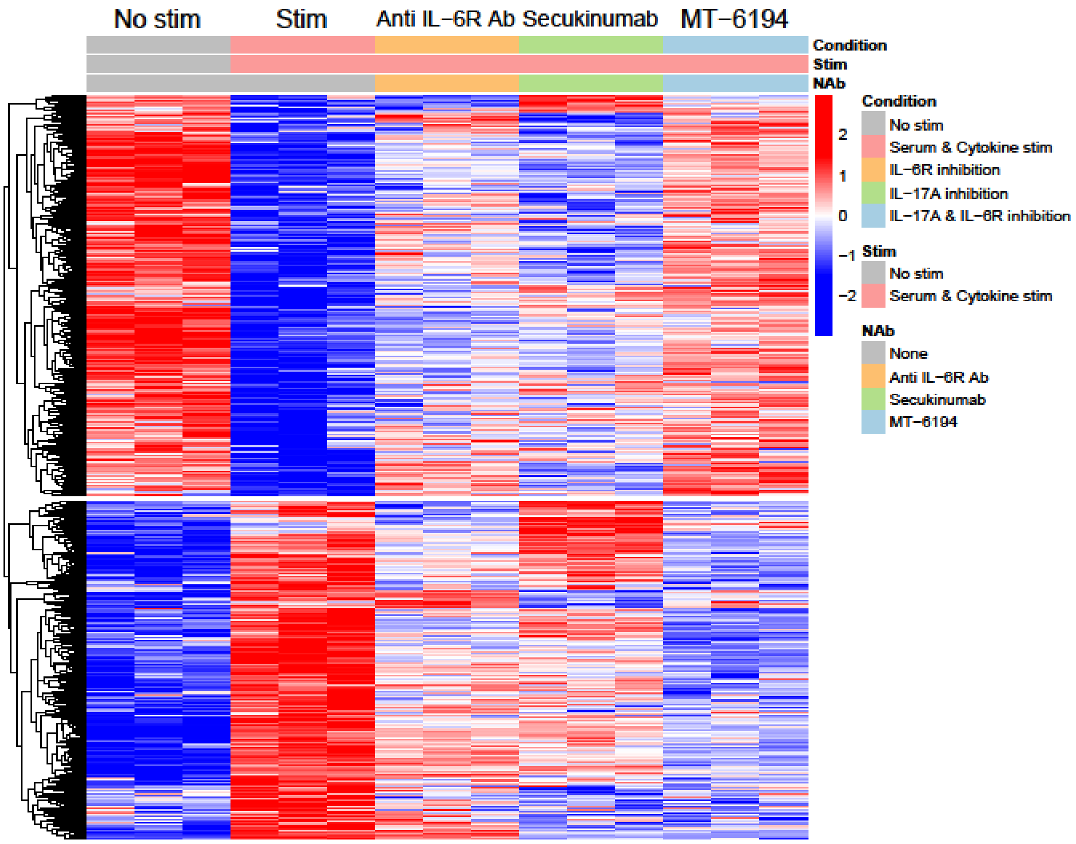

Supplementary Figure 5 Heatmap of the differential expressed genes in *in vitro* neutralizing experiment.

The heat map provides an overview of the gene expression changes caused by neutralizing antibodies. Genes showing significant changes in at least one neutralizing antibody were included. The expression level of each gene (or row) was scaled so that the mean expression level is was 0 and the standard deviation is was 1. The genes were rearranged by the hierarchical clustering (distance: “euclidean”; method: “completed”) method.

Characteristics	SSc (N = 57)	HCs (N=44)	P-value
Age (years)	60.0 (52.0-71.0)	43.5 (34.3-50.0)	<0.01
Female sex, n (%)	54 (94.7)	34 (77.3)	0.015

Supplementary Table 1. Ages and gender ratio of SSc patients and healthy controls (HC) in this study. Ages are expressed as the median with interquartile range (IQR) and statistical difference between SSc patients and HC was analyzed by Mann Whitney U-test. Difference of gender ratio (% of female) was analyzed by Fischer's exact test.
