# Supplementary figures and images for: Interleukin-17A is a potential therapeutic target predicted by proteomics for systemic sclerosis patients at high risk of pulmonary arterial hypertension
Source: Sci Rep. 2024 Nov 27;14:29484. doi: 10.1038/s41598-024-76987-6 (PMC11603215; doi:10.1038/s41598-024-76987-6)

Plasma protein, NPX

IL17A

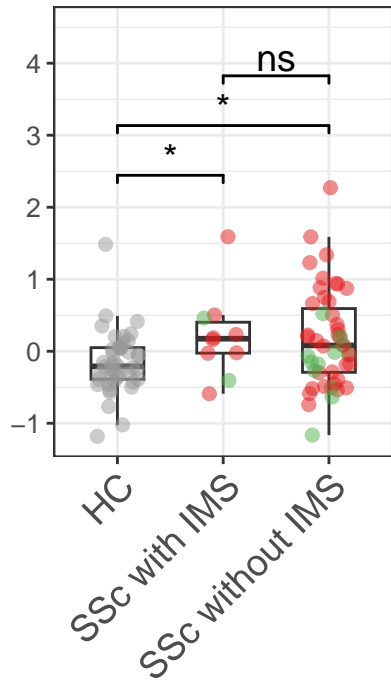

IL6

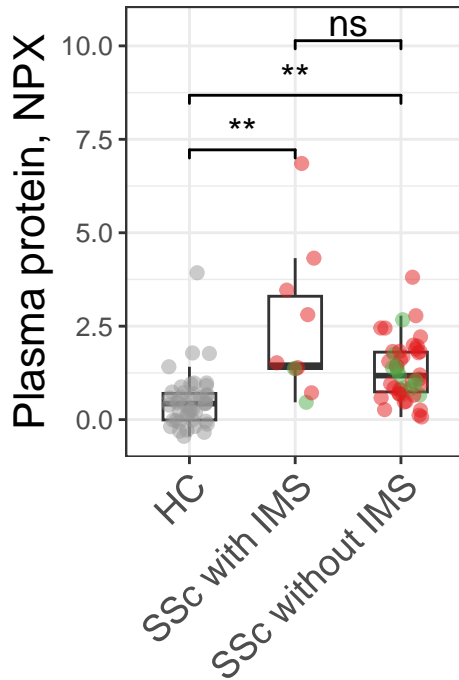

- HC
- FVC/DLCO < 1.6
- FVC/DLCO ≥ 1.6

Supplement: Supplementary file 9 — Supplementary Information 9. [file 41598_2024_76987_MOESM9_ESM.pdf]

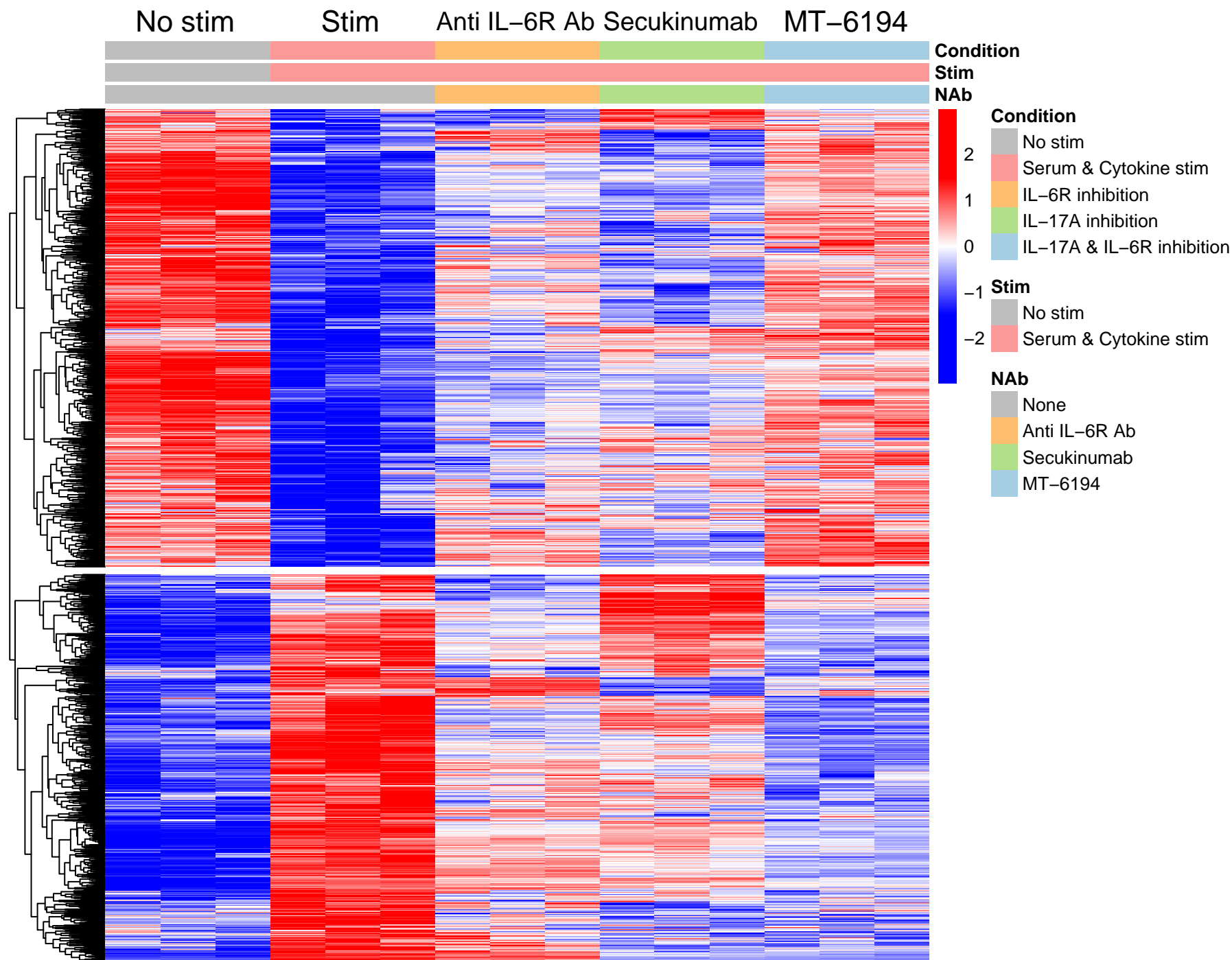

Supplement: Supplementary file 10 — Supplementary Information 10. [file 41598_2024_76987_MOESM10_ESM.pdf]
